# Open repair of subscapularis tendon tears leads to complete relief of symptoms in the majority of patients, but often fails to restore functional range of motion

**DOI:** 10.1177/17585732241249079

**Published:** 2024-04-24

**Authors:** Timon H Geurkink, Bart W Oudelaar, Celeste L Overbeek, Jorrit Jasper, Rob GHH Nelissen, Jochem Nagels

**Affiliations:** Department of Orthopaedics, 4501 Leiden University Medical Centre, Leiden, the Netherlands

**Keywords:** Subscapularis, subscapularis tears, open repair, rotator cuff

## Abstract

**Purpose:**

To investigate the outcome of open subscapularis (SSC) repair in terms of complete relief of symptoms, regaining functional range of motion ((ROM), i.e. minimum ROM to complete all tasks of daily living) and retear rate.

**Methods:**

Sixty-one patients who underwent open SSC repair between 2012 and 2019 were included in a retrospective cohort study. Primary outcome measures (complete relief of symptoms, obtaining functional ROM, SSC retears), were assessed at minimum 1-year follow-up. Prognostic factors for these outcome measures were identified.

**Results:**

At final follow-up, 44 patients (72%) reported complete relief of symptoms. Pre-operatively, 23 patients (40%) had a functional ROM, which increased to 33 patients (54%) post-operatively. Eight Patients (13%) had a retear after a median follow-up of 21 months (range: 3-35). Lafosse type IV tears were associated with having persisting symptoms (OR 5, 95 confidence interval (CI) 1.2–17.9, *p *= 0.024) and retears (OR 7, 95 CI 1.9–37.7, *p *= 0.031).

**Conclusion:**

The majority of patients reported complete relief of symptoms after SSC repair; however, only 54% obtained a functional ROM. Measuring outcome in terms of complete relief symptoms and regaining functional ROM is useful for the surgeon to evaluate the effect of surgical intervention and provides tangible information for patients.

## Introduction

The subscapularis (SSC) muscle is the largest and most powerful rotator cuff muscle and plays an integral part in shoulder stability and movement.^1^ Disruption of the SSC muscle results in altered glenohumeral biomechanics, which may lead substantial functional disability and pain.^2^ Despite its importance, tears of the SSC have received limited attention in orthopedic literature.^3^ SSC tears are, however, very common as the SSC is involved in 35–49% of all patients undergoing surgery for rotator cuff tears.^4–6^ Enhanced understanding of its importance in shoulder functionality has led to an increased insight on the importance of repairing SSC tears for both isolated and combined rotator cuff tears.^3,7–12^

Multiple studies on both open and arthroscopic repair of SSC tears have been conducted. Both techniques show comparable results in terms of relief of pain, improvement of range of motion (ROM) and healing rates.^2,13^ Studies investigating the outcome of SSC repair often only use shoulder scales (e.g. Constant-Murley score, ASES) to evaluate the effect of SSC repair. These scales, however, primarily rely on subjective measures particularly focusing on the pain and strength domains but offer limited information on overall shoulder function.^14,15^ Little is known about the effectiveness of SSC repair in terms of obtaining complete relief of symptoms and regaining functional ROM (i.e. the minimum required ROM to complete all tasks of daily living). These outcome scores are not only important for the surgeon to evaluate the effect of surgery, but are also easy to interpret and support informed shared decision-making and patient expectation management.^15^ We therefore asked: What are the outcomes of open SSC repair in terms of (1) complete relief of symptoms, (2) regaining functional ROM and (3) retear rate? Our secondary aim was to identify prognostic factors for these outcomes.

## Materials and methods

### Study design and participants

**Figure 1.**
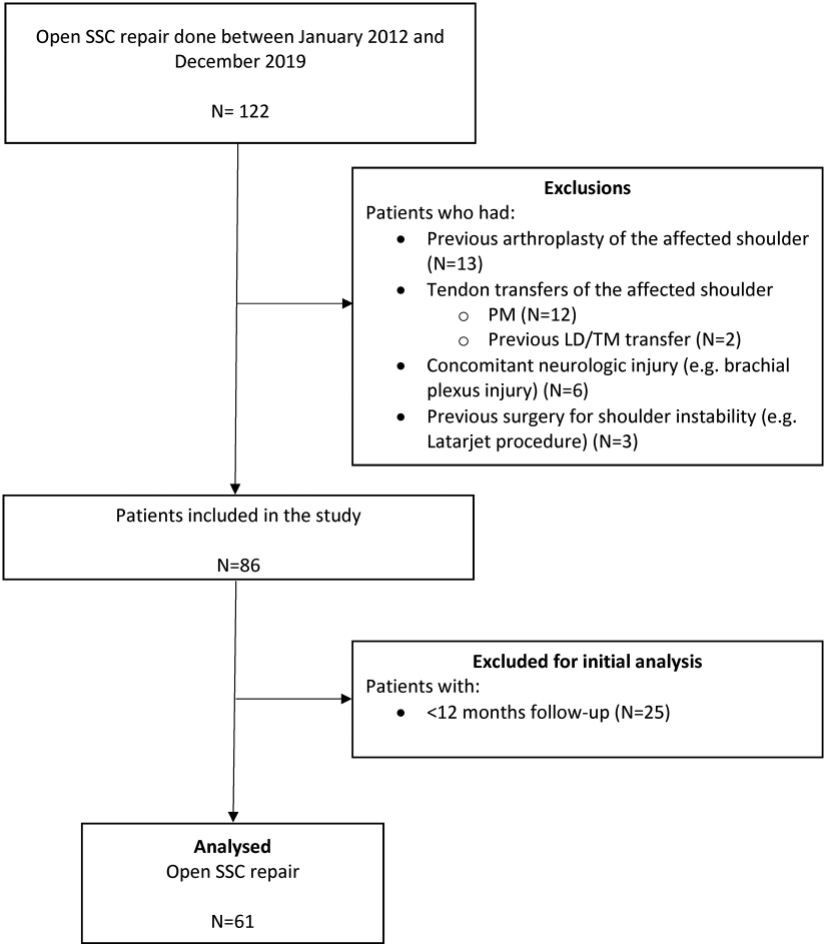
Flowchart. SSC = Subscapularis PM = Pectoralis major, LD = Latissimus Dorsi, TM = Teres Major.

This study was set up as a retrospective cohort study of all consecutive patients with a rotator cuff tear involving the SSC muscle operated by the senior authors (RN, JN) at the department of Orthopaedics, Leiden University Medical Center (LUMC, The Netherlands). After institutional medical ethical review board approval, data of 122 patients with a primary SSC repair between January 2012 and December 2019 were reviewed. All patients aged 18 + years with a primary open repair of the SSC tendon, a minimum follow-up of 12 months and pre-operative magnetic resonance imaging (MRI) were included. Exclusion criteria were previous arthroplasty surgery, Latarjet procedure, tendon transfers of the affected shoulder and concomitant neurologic injury (e.g. axillary nerve or brachial plexus injury). After applying the exclusion criteria, 86 patients were included in the study. Twenty-five patients (29%) were lost to follow-up before the 12-month follow-up was completed, leaving 61 patients available for analysis (Figure 1).

### Data collection, radiographic assessment and outcome measures

Individual patient records were independently evaluated by two of the authors (TG, BO) to collect demographics and clinical data. Active ROM of patients was evaluated pre-operatively and during follow-up using goniometry. The median final follow-up time was 17,0 months (range 12–105 months).

Pre-operative standard anteroposterior and axial radiographs were obtained to analyze presence of glenohumeral osteoarthritis (Kellgren-Lawrence classification).^16^ All patients had MRI of the shoulder prior to the intervention to determine the location and extent of the SSC rupture according to the Lafosse classification, the length of the SSC tendon (tendon elongation), the presence of fatty degeneration (Goutallier classification), SSC muscle atrophy (Thomazeau classification) and involvement of the supraspinatus (SS), infraspinatus (IS) tendons.^6,17–19^ The Kellgren-Lawrence score (0/1 vs 2-4), Lafosse (1-3 vs 4), Goutallier (0/1 vs 2-4) and Thomazeau (0/1 vs 2/3) were dichotomized for the purpose of statistical analysis.

At the final follow-up patients were asked whether they were symptom-free or whether they experienced persisting symptoms (complete relief yes/no). Persisting symptoms were defined as any symptom attributable to the torn SSC tendon that did not disappear after surgery such as ongoing shoulder pain, weakness or functional limitations in daily life. Furthermore, data on additional treatments and retears were collected. In all patients that were suspected of a retear the diagnosis was confirmed with MRI. Active physical ROM of the shoulder was evaluated pre-operative and at final follow-up. The latter was used as a composite measure for predefined functional ROM values as defined by Namdari et al.: 120° of forward elevation (FE), 130° of abduction (AB) and 60° of external rotation (ER) in 90° of abduction, i.e. the minimum required ROM to perform all functional tests of the Simple Shoulder Test, the University of Pennsylvania Shoulder Score (PSS) and the American Shoulder and Elbow Surgeon (ASES) score.^20^

#### Surgical procedure

Through a deltopectoral approach, with the patient in beach-chair position, the torn SSC tendon was identified, from the rotator cuff interval downward. The residual parts still fixed on the minor tubercle were released as well as any adhesions to the conjoined tendon, caudal surface of the coracoid process base and anterior side from the glenoid. Next, the tendon was reinserted to the lesser tubercle with two 5.0  mm bone anchors (Mitek Fastin RC with orthocord sutures, DePuy J&J, USA). Tenodesis of the tendon of the long head of the biceps brachii was performed when the tendon was still in continuity. After surgery, the shoulder was immobilized in a shoulder immobilizer (Mizuho OSI, Union City, USA) in internal rotation for 6 weeks, at 2 weeks active ROM exercises to a maximum of 70° of abduction and 0° of ER in adduction were initiated.

#### Statistical analysis

Parametric continuous data were described as means, standard deviation (SD) and 95% confidence intervals (CIs), and nonparametric data were expressed as median and interquartile range. Categorical data were presented by numbers and percentages. Pre- and post-operative scores were compared using the Wilcoxon rank-sum or McNemar test.

Univariate analyses (independent t-tests or the Mann–Whitney U tests for continuous data and chi-squared or Fisher exact test for categorical data) were performed to select factors to enter in the multivariate logistic regression analysis. Factors that approached a correlation of *p* < 0.10 were included for multivariate logistic regression analysis using complete case analysis.

Stepwise regression through backward elimination was performed to determine prognostic factors associated with complete relief of symptoms (complete relief yes/no) at final follow-up, retear yes/no and obtaining functional ROM. A two-sided significance level of *p* < 0.05 was applied during the process of elimination. All statistical analyses were performed using the statistical package SPSS version 20.0 (IBM, Armonk, NY, USA).

## Results

Baseline characteristics of the patients are presented in Table 1. Twenty-two (36%) patients had previous surgery of other tendons of the affected shoulder. An isolated SSC tear was present in 23% (n = 14), an anterosuperior tear (SSC + SS) was seen in 41% (n = 25), while 36% (n = 22) had a combined tear of the SSC, SS, and IS muscles.

**Table 1.** Patient demographics

Baseline characteristics (n = 61)	
Male, no. (%)	**36 (59%)**
Age, mean (SD) yrs	**61 (8.4)**
Duration of symptoms, median (IQR), mo	**14.6 (7.5-26.6)**
ASA	
_1, No. (%)	**19 (31%)**
_2, No. (%)	**35 (57%)**
_3, No. (%)	**7 (12%)**
BMI, mean (SD), kg/m^2^	**27.7 (4.8)**
Rheumatic disease, no.(%)	**3 (5%)**
Diabetes mellitus, no. (%)	**10 (16%)**
Previous surgery (%)	**22 (36%)**
_Supraspinatus repair, no. (%)	**18 (30%)**
_Subacromial decompression, no. (%)	**5 (8%)**
_Capsular shift, no. (%)	**2 (3%)**
Kellgren-Lawrence grade (N = 59)	
_0, no. (%)	**42 (69%)**
_1, no. (%)	**15 (25%)**
_2, no. (%)	**2 (3%)**
Length tendon in mm, mean (SD) (N = 59)	**51.9 (10.4)**
Retraction tendon in mm, mean (SD)	**22.0 (13.6)**
Lafosse tear type	
_1. no. (%)	**5 (8%)**
_2, no. (%)	**20 (33%)**
_3, no. (%)	**14 (23%)**
_4, no. (%)	**22 (36%)**
Goutallier grade (n = 49)	
_0, no. (%)	**6 (10%)**
_1, no. (%)	**21 (34%)**
_2, no. (%)	**15 (25%)**
_3, no. (%)	**6 (10%)**
_4, no. (%)	**1 (2%)**
Thomazeau grade (N = 50)	
_0, no. (%)	**27 (44%)**
_1, no. (%)	**14 (23%)**
_2, no. (%)	**9 (15%)**
Tear type	
_Isolated SSC, no. (%)	**14 (23%)**
_SSC + SSP, no. (%)	**25 (41%)**
_SSC + SSP + ISP, no. (%)	**22 (36%)**

SSC = Subscapularis, SSP = Supraspinatus, ISP = Infraspinatus, SD = standard deviation, yrs = years, mo = months, BMI = Body mass index, mm = millimeter.

### Complete relief of symptoms

At median final follow-up (17,0 months; range 12–105), 72% (n = 44) of patients reported complete relief of symptoms. Seventeen (28%) patients required additional treatments, such as subacromial (n = 7) and/or acromioclavicular joint (n = 13) corticosteroid injections (Kenacort-A 10). Overall, three (5%) complications occurred: one patient had an SSC tendon anchor failure, requiring revision surgery and reattachment of the SSC and two patients had a frozen shoulder post-operatively, which resolved during study course.

### Range of motion

Post-operative, the ROM improved in all of the movement planes of the shoulder (Table 2). Pre-operatively, functional forward flexion, abduction or ER in 90 degrees of abduction was seen in, respectively, 53% (n = 32), 41% (n = 25) and 62% (n = 38) of patients, and 40% (n = 23) of patients had a complete functional ROM. Post-operatively, 75% (n = 45), 56% (n = 34) and 82% (n = 50) of patients had a functional forward flexion, abduction or ER in 90 degrees of abduction, and a complete functional ROM was seen 54% (n = 33) of patients.

**Table 2.** Active range of motion of the shoulder and lift-off test.

	Pre-operative	% Functional	Post-operative	% Functional	*p*-value
FE	121 (51)	53%	139 (39)	75%	*0.019*
AB	106 (55)	41%	127 (42)	56%	*0.005*
ER	61 (26)	62%	70 (23)	82%	*0.044*
Complete functional ROM		40%		54%	
Positive lift-off test	80,3%		19,7%		*<0.001*

Active Ranges of Motion are presented in degrees. Predefined functional ROM values: 120° of forward elevation, 130° of abduction and 60° of external rotation in 90° of abduction. The values are given as the mean and SD; SD = Standard deviation; FE = Forward elevation; AB = Abduction; ER = External rotation in 90 degrees of abduction; ROM=range of motion.

### Retear

Eight (13%) patients had a retear of the SSC tendon after a median of 21 months (range 3–35). Seven patients received a pectoralis major tendon transfer accordingly.

### Prognostic factors

The results of the univariate analyses for factors related to complete relief of symptoms, reaching a functional ROM and for retear are presented in Appendix A. No significant differences were seen between patients with isolated anterosuperior and combined tears with regard to primary outcome measures. (Appendix A). Patients with a complete (Lafosse type IV) tear had higher odds for complete relief of symptoms (OR 4.7, 95 CI 1.2–17.9, *p* = 0.024) and were more likely to develop a retear (OR 6.7, 95 CI 1.9–37.7, *p* = 0.031). Male sex was associated with higher odds (OR 3.2, 95 CI 1.0–10.1, *p* = 0.049) for reaching a functional ROM post-operatively (Table 3).

**Table 3.** Outcomes logistic regression

Outcome	Factor	Indicator	OR (95% CI)	*p*-value
Complete relief	Lafosse 1-3	Lafosse 4	4.7 (1.23 to 17.87)	**0**.**024**
	Pre-op duration symptoms (months)		1.02 (1.00. to 1.04)	0.087
Functional ROM	Male Pre-op duration symptoms (months)	Female	3.2 (1.003 to 10.11)	**0**.**049**
			0.99 (0.97 to 1.01)	0.221
Retear	Lafosse 4	Lafosse 1-3	6.7 (1.19 to 37.71)	**0**.**031**

Bold values indicates statistical significance of p < 0.05. ROM=range of motion; CI=confidence interval.

## Discussion

This study demonstrated that 72% patients following open repair of SSC tears report complete relief of symptoms. Post-operatively, the active ROM in FE, abduction and ER increased significantly, but as a composite measure of active motion in three planes, a functional ROM was only obtained in 54% of patients. In 13% a lasting repair of the SSC failed, as these patients had retears of the SSC tendon. Retears occurred at a median of 21 months and were more likely to occur in patients with complete (Lafosse type IV) tear of the SSC.

Our study has some limitations: first, the retrospective study design which comes with inherent flaws such as no prospective data collection and missing data. For that matter, 25 patients were lost to follow-up at the 12-month period. In depth analysis between the included and lost to follow-up group, however, did not show any significant differences between both groups. Secondly, muscle strength was not measured during follow-up. Therefore, standardized outcome measures, such as the Constant-Murley score could not be calculated, making it more difficult to compare our results with the existing literature. Lastly, a substantial part of the patients received previous surgery of the affected shoulder, which might influence the results of the present study. Possibly, these patients already had a SSC tear at first presentation, which might have been missed initially. Unfortunately, we could not investigate this as these patients received treatment in different institutions.

Open repair of the SCC was first published by Hauser in 1954.^21^ Since then, several studies were published on both open and arthroscopic repair of SSC tears, both techniques showing comparable results.^2,13,22^ To date, the largest study on the efficacy of open repair of SSC tears is by Edwards et al. who analyzed the outcome of open repair of isolated SSC tears in 84 shoulders.^23^ After a mean follow-up of 45 months, a significant improvement of Constant scores was found and, subjectively, 45 patients (54%) reported an excellent result and 30 patients (36%) a good result. Other studies on open SSC repair for both isolated and anterosuperior (SSC + SS) tears show similar results with patient satisfaction rates ranging between 42 to 98%.^24,25^ Comparable results after open repair of SSC were found in our study as 72% of patients reported complete relief of symptoms.

Most studies, investigating the effectiveness of open SCC repair, evaluate shoulder function using physician measured outcome scales such as the Constant-Murley score. Despite its wide acceptance and frequent use, the validity of such scales has been questioned as they do not adequately address specific shoulder pathology, include subjective measures and score on the pain and strength domains particularly with limited value on overall shoulder function.^14,26,27^ Therefore, it has been suggested to evaluate the effect of surgical intervention by measuring an objective measure of functional outcome: the functional ROM.^15^ The latter is also easy to interpret by surgeons and important for patient expectation management. Namdari et al. analyzed the overall shoulder motion required to perform a variety of functional tasks of daily living and concluded that to successfully complete all tasks of daily living, approximately 120° of FE, 130° of abduction and 60° of ER in 90° abduction is needed, which is considerably less than a full ROM.^20^

In this study, 40% of patients had a functional ROM prior to repair of the SSC tendon, which increased to 54% post-operatively at the last follow-up. This study is the first to evaluate the effectiveness of open repair of the SCC in terms of regaining a functional ROM and to describe such a remaining functional deficit. In previous studies, only Bartl et al. described a residual rotator cuff strength deficit in patients receiving open SSC repair, while few other studies described post-operative subscapular dysfunction in terms of persistent positive lift-off tests.^8,23,28^ So, despite previously published good results on physician measured outcome score, like the Constant score, results of the present study demonstrate that open SSC repair fails to restore functional ROM in almost half of the patients indicating that many patients will keep some extend of functional impairment.

In this study, 13% of patients had a retear after a median duration of 21 months. Comparable retear rates after both open and arthroscopic repair were found by others ranging from 4 to 35%.^7,24,29^ This large variation in retears is probably due to the heterogeneity in patients and SSC tear characteristics.

Previous studies have identified several prognostic factors for the outcome of both open and arthroscopic SSC repair. The present study found that patients with a Lafosse type IV tears were less likely to report complete relief of symptoms and that male patients were more likely to reach a functional ROM. Furthermore, Lafosse type IV tear of the SSC muscle significantly increased the chance of retear compared to Lafosse type I-III tears. These results are in concordance with other studies which demonstrated a larger tear size, tendon retraction, fatty degeneration, older age, female sex and a longer time from symptom onset to surgery are associated with less favorable outcomes.^7,10,29–32^

A substantial part of the included patients had a combined SSC tear with either the SS (41%) or SS and IS muscles (36%). In all combined cases, only open repair of the SSC tendon was performed. Results of this study demonstrate that involvement of the SS and/or IS tendons does not affect the outcome. Repair of only the SSC tendon in massive (SS + IS + SSC) cuff tears has only been evaluated previously by Austin et al. who concluded that arthroscopic isolated SSC repair is a promising and safe method for treating massive cuff tears.^12^ Isolated repair of SSC tendons in patients with massive rotator cuff tears seems therefore to be a suitable option.

In conclusion, results of this study demonstrate that open repair of SCC tears results in complete relief of symptoms in the majority patients, but is less effective in terms of regaining functional ROM. Measuring the effectiveness in terms of complete relief of symptoms and a composite functional ROM may have important implications during the pre-operative consultation as patients should be provided with reasonable expectations regarding improvement in function and are useful to evaluate treatment effect.

## Supplemental Material

sj-docx-1-sel-10.1177_17585732241249079 - Supplemental material for Open repair of subscapularis tendon tears leads to complete relief of symptoms in the majority of patients, but often fails to restore functional range of motion Supplemental material, sj-docx-1-sel-10.1177_17585732241249079 for Open repair of subscapularis tendon tears leads to complete relief of symptoms in the majority of patients, but often fails to restore functional range of motion by Timon H Geurkink, Bart W Oudelaar, Celeste L Overbeek, Jorrit Jasper, Rob GHH Nelissen and Jochem Nagels in Shoulder & Elbow
