# Supplementary material for: Open repair of subscapularis tendon tears leads to complete relief of symptoms in the majority of patients, but often fails to restore functional range of motion
Source: Shoulder Elbow. 2024 Apr 24;17(2):166–72. doi: 10.1177/17585732241249079 (PMC11571163; doi:10.1177/17585732241249079)
Supplement: sj-docx-1-sel-10.1177_17585732241249079 - Supplemental material for Open repair of subscapularis tendon tears leads to complete relief of symptoms in the majority of patients, but often fails to restore functional range of motion [file sj-docx-1-sel-10.1177_17585732241249079.docx]

| **Appendix A: univariate analyses for factors related to complete relief of symptoms (yes/no), retear (yes/no) and reaching a functional ROM (yes/no)** | | | | | | |
| --- | --- | --- | --- | --- | --- | --- |
| **Factor** | **Complete relief (yes)** | **p-value** | **Retear (yes)** | **p-value** | **Functional ROM (yes)** | **p-value** |
| Sex (male/female; %) | 75%/68% | 0.549 | 11%/16% | 0.706 | 64%/42% | **0.090** |
| Age (mean difference (95% CI) | -0,7 (-5.5 to 4.1) | 0.775 | -0.7(-5.8 to 4.4) | 0.822 | 1.4 (-3.0 to 5.9) | 0.520 |
| Duration of symptoms (mean difference (95% CI); mo) | -36,6 (-95,6 to 22,2) | **0.068** | 70.2 (-82,7 to 223,1) | 0.576 | -18,5 (-64,4 to 11,3) | **0.075** |
| Traumatic onset (yes/no; %) | 75%/68% | 0.493 | 9%/18% | 0.451 | 61%/48% | 0.335 |
| ASA (1 vs. 2/3) | 84%/67% | 0.157 | 0%/19% | **0.049** | 58%/54% | 0.759 |
| BMI | 1.2 (-1.5 to 3.9) | 0.378 | -1.6 (-5.2 to 2.1) | 0.389 | -1.4 (-3.9 to 1.0) | 0.254 |
| Comorbidities |  |  |  |  |  |  |
| _Rheumatic disease (yes/no; %) | 67%/72% | 1.000 | 33%/12% | 0.349 | 0%/58% | **0.085** |
| _Diabetes mellitus (yes/no; %) | 80%/71% | 0.711 | 0%/16% | 0.332 | 70%/52% | 0.488 |
| Previous surgery (yes/no; %) | 64%/77% | 0.266 | 5%/18% | 0.239 | 55%/55% | 0.957 |
| Radiologic factors |  |  |  |  |  |  |
| _Kellgren-Lawrence grade (0/1 vs. 2-4) | 72%/50% | 0.497 | 12%/50% | 0.255 | 55%/50% | 1.000 |
| _Length tendon (mean difference (95% CI); mm) | 1.0 (-5.2 to 7.1) | 0.753 | 4.9 (-3.0 to 12.8) | 0.216 | 5.0 (-0.4 to 10.6) | **0.068** |
| _Retraction tendon (mean difference (95% CI); mm) | 2.8 (-5.0 to 10.7) | 0.473 | 4.3 (-6.1 to 14.7) | 0.408 | 0.0 (-7.0 to 7.0) | 0.994 |
| _Lafosse tear type (1-3 vs. 4) | 80%/59% | **0.088** | 5%/27% | **0.021** | 54%/57% | 0.807 |
| _Goutallier grade (0/1 vs. 2-4) | 67%/73% | 0.647 | 11%/23% | 0.440 | 52%/57% | 0.715 |
| _Thomazeau grade (0/1 vs. 2/3) | 71%/67% | 1.000 | 10%/44% | **0.026** | 56%/50% | 1.000 |
| Tear type |  |  |  |  |  |  |
| _Isolated SSC | 71% | 0.996 | 14% |  | 64% |  |
| _Anterosuperior (SSC+SSP;) | 72% |  | 4% | 0.169 | 56% | 0.631 |
| _SSC+SSP+ISP | 73% |  | 23% |  | 48% |  |

**Appendix A. Results univariate analysis**
SSC=Subscapularis, SSP = Supraspinatus, ISP= Infraspinatus, CI= confidence interval, yrs = years, mo = months, BMI = Body mass index, mm = millimeter
